# Perfusion and Metabolic Neuromonitoring during Ventricular Taps in Infants with Post-Hemorrhagic Ventricular Dilatation

**DOI:** 10.3390/brainsci10070452

**Published:** 2020-07-15

**Authors:** Ajay Rajaram, Lawrence C. M. Yip, Daniel Milej, Marianne Suwalski, Matthew Kewin, Marcus Lo, Jeffrey J. L. Carson, Victor Han, Soume Bhattacharya, Mamadou Diop, Sandrine de Ribaupierre, Keith St. Lawrence

**Affiliations:** 1 Imaging Program, Lawson Health Research Institute, London, ON N6A 4V2, Canada; lawrence.yip@uwo.ca (L.C.M.Y.); dmilej@uwo.ca (D.M.); msuwalsk@uwo.ca (M.S.); mkewin2@uwo.ca (M.K.); mlo53@uwo.ca (M.L.); jcarson@lawsonimaging.ca (J.J.L.C.); mdiop@lawsonimaging.ca (M.D.); kstlaw@lawsonimaging.ca (K.S.L.); 2 Department of Medical Biophysics, Schulich School of Medicine and Dentistry, Western University, London, ON N6A 3K7, Canada; sandrine.deribaupierre@lhsc.on.ca; 3 Division of Neonatal-Perinatal Medicine, Department of Pediatrics, London Health Sciences Centre, London, ON N6A 3K7, Canada; victor.han@lhsc.ca (V.H.); Soume.Bhattacharya@lhsc.on.ca (S.B.); 4 Department of Clinical Neurological Sciences, London Health Sciences Centre, London, ON N6A 5W9, Canada

**Keywords:** near-infrared spectroscopy, diffuse correlation spectroscopy, premature brain injury, hydrocephalus, intraventricular hemorrhaging, ventricular tap, neuromonitoring, cerebral blood flow, metabolism, cytochrome c oxidase

## Abstract

Post-hemorrhagic ventricular dilatation (PHVD) is characterized by a build-up of cerebral spinal fluid (CSF) in the ventricles, which increases intracranial pressure and compresses brain tissue. Clinical interventions (i.e., ventricular taps, VT) work to mitigate these complications through CSF drainage; however, the timing of these procedures remains imprecise. This study presents Neonatal NeuroMonitor (NNeMo), a portable optical device that combines broadband near-infrared spectroscopy (B-NIRS) and diffuse correlation spectroscopy (DCS) to provide simultaneous assessments of cerebral blood flow (CBF), tissue saturation (S_t_O_2_), and the oxidation state of cytochrome c oxidase (oxCCO). In this study, NNeMo was used to monitor cerebral hemodynamics and metabolism in PHVD patients selected for a VT. Across multiple VTs in four patients, no significant changes were found in any of the three parameters: CBF increased by 14.6 ± 37.6% (*p* = 0.09), S_t_O_2_ by 1.9 ± 4.9% (*p* = 0.2), and oxCCO by 0.4 ± 0.6 µM (*p* = 0.09). However, removing outliers resulted in significant, but small, increases in CBF (6.0 ± 7.7%) and oxCCO (0.1 ± 0.1 µM). The results of this study demonstrate NNeMo’s ability to provide safe, non-invasive measurements of cerebral perfusion and metabolism for neuromonitoring applications in the neonatal intensive care unit.

## 1. Introduction

Premature birth, defined as a gestational period less than 37 weeks, occurs in 8% of infants in Canada [1]. Those born with lower birthweights (<1500 g) are at an increased risk of neurological complications, including deficits in cognition and behaviour as well as a higher incidence of major motor disorders (e.g., cerebral palsy) [2]. The largest contributor to preterm brain injury is intraventricular hemorrhaging (IVH). The pathogenesis of IVH involves the combination of an immature cerebral vascular anatomy (i.e., a highly vascularized germinal matrix) and poor cerebral blood flow (CBF) regulation [3,4]. It is believed that IVH elicits an inflammatory response, followed by fibrosis and subsequent obstruction of cerebral spinal fluid (CSF) drainage [5]. This can lead to accumulation of CSF, increased intracranial pressure (ICP), and dilatation of the entire ventricular system, referred to as post-hemorrhagic ventricular dilatation (PHVD).

PHVD is a major complication in severe cases of IVH (Grade III and higher), where more than 50% of the lateral ventricles are experiencing a bleed [6,7]. Due to a progressively increasing ICP, any delay in treatment can result in white matter atrophy and neurologic deterioration [4,8]. Cranial ultrasound (cUS) is the current gold standard in diagnosing and monitoring the progression of PHVD through ventricular measurements such as the ventricular index (VI), anterior horn width (AHW), and thalamo-occipital distance (TOD) [9]. Given these measures, there is still some debate over the timing of clinical intervention [10]. An early approach used by many European centres relies on ventricular measurements before patients are symptomatic. Intervention consists of a variety of techniques to drain CSF, including external ventricular drains (EVD), lumbar punctures (LP), and ventricular taps (VT), among others. [11]. In comparison, many North American centres employ a later approach where treatment is initiated depending on the ventricular index and clinical signs (e.g., increased ICP, head circumference, ventricle size, tense fontanelle, etc.) [12,13]; similar techniques are then implemented to drain CSF.

The discrepancy in the timing of clinical intervention is largely due to a lack of adequate monitoring techniques to quantify PHVD progression and treatment efficacy. Recent work has elucidated the benefits of hemodynamic and metabolic neuromonitoring surrounding the development of PHVD [14,15]. These metrics have shown potential to act as prognostic markers of PHVD symptoms and could better indicate when to implement CSF drainage strategies. Measurements of CBF by transcranial doppler (TCD) have proven robust in premature infants; however, continuous monitoring is not ideal as TCD is operator-dependent and only measures macrovascular changes in blood flow [16,17]. Biomedical optics instead provide a safe, non-invasive technique ideal for longitudinal brain monitoring at the bedside [3,18,19,20].

This study presents an in-house built brain monitoring system referred to as NNeMo (Neonatal NeuroMonitor) that is capable of simultaneous measurement of cerebral perfusion, tissue saturation, and oxygen metabolism at the bedside through combining two optical techniques. Broadband near-infrared spectroscopy (B-NIRS) uses near-infrared light to measure absorption properties of tissue that can be used to estimate the concentrations of oxy and deoxyhemoglobin, from which the cerebral tissue saturation (S_t_O_2_) can be calculated [21]. B-NIRS can also provide a direct measurement of cerebral metabolism by monitoring absorption changes in the oxidation state of cytochrome c oxidase (oxCCO), which is directly related to mitochondrial ATP production in the electron transport chain [22]. Diffuse correlation spectroscopy (DCS) is an emerging technique that provides continuous monitoring of CBF by analyzing dynamic light scatter from red blood cells [23]. DCS has been validated against multiple modalities, including dynamic contrast-enhanced NIRS, Positron Emission Tomography with oxygen-15 (^15^O) labelled water, and Arterial spin labelling MRI [24,25,26]. NNeMo combines B-NIRS and DCS technology to simultaneously monitor CBF, S_t_O_2_, and oxCCO.

This study presents the clinical translation of NNeMo to monitor PHVD infants in the neonatal intensive care unit (NICU) undergoing CSF drainage via ventricular taps. Previous work using contrast-enhanced NIRS reported a small increase in CBF after the VT, but with no concurrent change in cerebral energy metabolism [14]. However, only single time-point measurements before and after the procedure were acquired due to the need to inject a contrast agent (indocyanine green). Here, we present continuous bedside monitoring of cerebral perfusion and metabolism in PHVD patients undergoing a VT, with the goal of assessing any potential treatment-related changes in CBF and oxCCO. Based on the magnitude of CBF changes previously reported [14], it was hypothesized that CSF drainage would lead to a modest increase in CBF, but with minimal impact on oxCCO.

## 2. Materials and Methods

### 2.1. Patient Population

This study was approved by the Western University Health Sciences Research Ethics Board (Project Identification Code: 17828), which adheres to the guidelines of the Canadian Tri-Council Policy Statement: Ethical Conduct for Research Involving Humans. Patients with PHVD were enrolled after obtaining parental consent. The initial IVH was diagnosed by cranial ultrasound (cUS) and graded according to the Papile scale [5]. The decision to perform a ventricular tap was based on standard of care, including clinical evidence (apnea, bradycardia, full fontanelles) and increased ventricle size as measured by cUS.

### 2.2. Study Design

**Figure 1:**
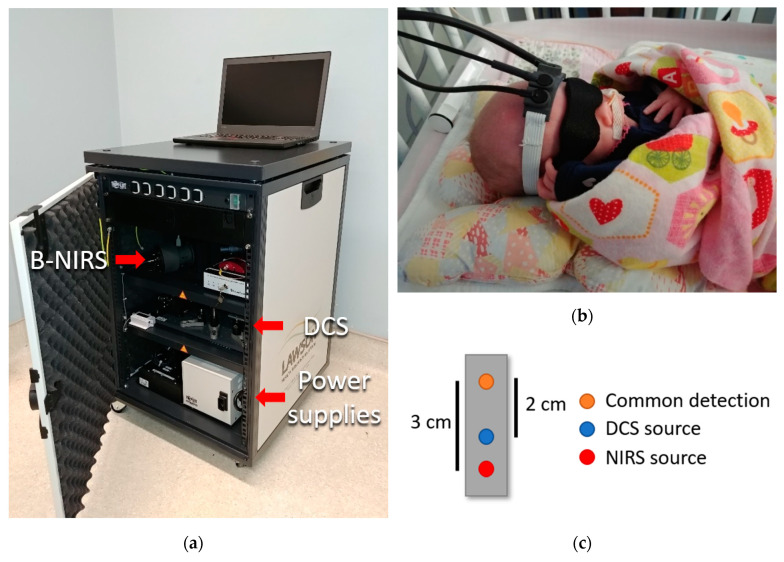
(**a**) Neonatal NeuroMonitor (NNeMo) combining broadband near-infrared spectroscopy (B-NIRS) and diffuse correlation spectroscopy (DCS) techniques. (**b**) Optical fibers affixed to an infant’s head using a 3D-printed probe holder. A phototherapy eye shield was used as a safety precaution. (**c**) Optical fiber schematic showing two optical sources and a common detection fiber (SDD: DCS 2 cm; B-NIRS 3 cm).

Infants receiving a VT were monitored throughout the procedure with NNeMo (Figure 1a). Prior to surgical setup, optical probes were placed on the infant’s scalp above the frontoparietal cortex and held in place by a custom-built probe holder (Figure 1b). Two optical probes (one for the B-NIRS and one for the DCS) delivered light to the scalp. Source power levels were maintained within ANSI standards for skin exposure. A common detection fiber bundle was used to collect diffusely reflected light that had propagated through the head. The DCS and B-NIRS systems collected light at source-detector distances (SDD) of 2 and 3 cm, respectively, to ensure adequate brain interrogation (Figure 1c). As an additional precaution, a phototherapy eye shield was employed to avoid exposing the subject’s eyes to the light sources.

**Figure 2:**
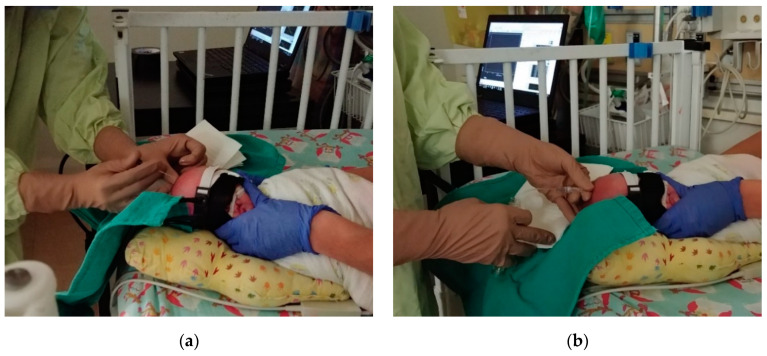
(**a**) Pediatric neurosurgeon inserting a needle into the ventricle and (**b**) draining cerebral spinal fluid (CSF) from the patient. Optical probes were affixed to the patient’s head throughout the procedure.

Following sterilization of the surgical site, a VT was performed by a pediatric neurosurgeon. A needle was inserted into the lateral ventricles (Figure 2a) to allow for CSF drainage (Figure 2b). Following sufficient CSF removal, as determined by the clinician, the needle was removed and gentle pressure was applied to the needle insertion site. Optical data were collected throughout the VT, extending a minimum of 10 min prior to and following the procedure. B-NIRS and DCS data were acquired sequentially at a sampling rate of 250 ms and each dataset was averaged to achieve a final temporal resolution of 7 s.

### 2.3. Instrumentation

NNeMo was constructed by combining B-NIRS and DCS modalities using a multiplexing shutter system controlled by custom software developed in LabView (2017 SP1, National Instruments, Austin, TX, USA) and MATLAB (R2018b, MathWorks, Natick, MI, USA) [27]. The acquisition times of both the B-NIRS and DCS were set to 250 ms (4 Hz). The shutter system cycled between the two subsystems in 3-s intervals. A total of 12 data points were acquired in each interval and averaged for both B-NIRS and DCS measurements. A 0.5-s delay was added between the intervals to account for shutter transition times and to avoid overlap between the B-NIRS and DCS data.

The B-NIRS light source was a 20-W halogen bulb (Hl-2000-HP, Ocean Optics, Delay Beach, FL, USA) that was filtered from 600 to 1000 nm and coupled into a custom optical fiber bundle (2.4 mm diameter, 30 µM core, 0.55 numerical aperture ((NA), Loptek, Berlin, Germany) that directed the light to the head. Light from the interrogated tissue was collected by 3 fiber bundles (2 mm diameter, 30 µm core, 0.55 NA, Loptek, Berlin, Germany) that were linearly aligned at the entrance of the spectrometer (iDus 420, Andor, Oxford Instruments, Abingdon, UK; 548–1085 nm bandwidth; 1.65 nm resolution; P&P Optica, Waterloo, ON, Canada). For DCS, light from a long coherence laser (DL785-100s, CrystaLaser, Reno, NV, USA) was coupled into a fiber bundle (4 × 200 µm core diameter, 0.22 NA, Loptek, Berlin, Germany) and directed towards the head. Light was collected by 4 single-mode fibers (8 µm core diameter, 0.12 NA, Loptek, Berlin, Germany) and coupled to a four-channel single photon counting module (SPCM-AQR-15-FC, Excelitas Technologies, Montreal, QC, Canada). The output from each detector was fed into an edge-detecting counter on a PCIe-6612 counter/timer data acquisition board (National Instruments) [28]. Photon counts were recorded and processed using in-house developed software (LabVIEW, MATLAB) [29]. For each detector, the software generated intensity autocorrelation curves at 50 delay times ranging from 1 µs to 1 ms.

For use in the NICU, the optical fibers were designed to be lightweight and flexible, with a 90° bend at the patient end to further minimize weight on the infant. A 3D-printed fiber probe holder (5 × 2 × 1 cm; Flexible Resin, Form 2, Formlabs, Somerville, MA, USA) was designed to secure the three optical probes (2 emission, 1 detection) to the patient’s forehead using a soft and flexible base material with a non-abrasive strap (Figure 1b).

### 2.4. Data Processing

#### 2.4.1. NIRS Analysis: Quantifying S_t_O_2_ and Changes in oxCCO Concentration

Prior to clinical measurements, a reference spectrum was acquired using a pinhole attenuator to characterize the spectral properties of the optical system. In addition, a measurement of the dark noise was acquired to characterize ambient light conditions. For the clinical NIRS data, each reflectance spectrum, *R*(*λ*), was corrected for the reference and dark count spectra as follows:

(1) $$R\left(\lambda \right)=log_{10}\left(\frac{spectrum_{\lambda }-dark_{\lambda }}{reference_{\lambda }-dark_{\lambda }}\right),$$

where *spectrum_λ_* refers to the clinical measurement at wavelength *λ*.

To quantify chromophore concentrations, while accounting for the contribution of light scatter, a derivative approach was utilized [18,30,31]. First and second derivatives of *R*(*λ*) were calculated and fit to the solution of the diffusion approximation for a semi-infinite homogeneous medium [19]. Light absorption (Equation (2)) and scattering (Equation (3)) parameters were input into the solution as follows [30]:

(2) $$\mu_{a}\left(\lambda \right)=WF\cdot \varepsilon_{H_{2}O}\left(\lambda \right)+Hb^{b}\cdot \varepsilon_{Hb}\left(\lambda \right)+HbO_{2}^{b}\cdot \varepsilon_{HbO_{2}}\left(\lambda \right),$$

where *WF* is the tissue water fraction, *Hb^b^* and *HbO_2_^b^* are baseline concentrations of deoxyhemoglobin and oxyhemoglobin in µM, and *ε* refers to the unique extinction coefficient for each chromophore.

(3) $${\mu_{s}}^{\prime }=A{\left(\frac{\lambda }{800\left(\mathrm{nm}\right)}\right)}^{-\alpha },$$

where α is the scattering power and A is the *μ_s_*’ value at *λ* = 800 nm. Reflectance spectra measured at baseline were analyzed with a multi-parameter fitting algorithm based on a constrained least-square minimization using a custom MATLAB function (fminsearchbnd). Briefly, second derivative *R(λ)* spectra were fit by the model between 815 and 845 nm to obtain *WF*. The *WF* value was incorporated into the second derivative fit from 680 to 800 nm to determine *Hb^b^*. Lastly, *WF* and *Hb^b^* were used in the analysis of the first derivative spectrum. The fitting procedure extended from 680 to 845 nm to obtain an estimate of *HbO_2_^b^* [30].

Following baseline analysis, a modified Beer–Lambert law based on the UCLn algorithm (Equation (4)) was used to calculate changes in hemoglobin and oxCCO concentrations for the duration of the study [22].

(4) $$\left[\begin{array}{c} \Delta HbO_{2}\\ \Delta Hb\\ \Delta oxCCO \end{array}\right]=\frac{1}{DP}{\left[\begin{array}{ccc} \varepsilon_{HbO_{2}}\left(\lambda_{1}\right) & \varepsilon_{Hb}\left(\lambda_{1}\right) & \varepsilon_{oxCCO}\left(\lambda_{1}\right)\\ \vdots & \vdots & \vdots \\ \varepsilon_{HbO_{2}}\left(\lambda_{n}\right) & \varepsilon_{Hb}\left(\lambda_{n}\right) & \varepsilon_{oxCCO}\left(\lambda_{n}\right) \end{array}\right]}^{-1}\times \left[\begin{array}{c} \Delta A\left(\lambda_{1}\right)\\ \vdots \\ \Delta A\left(\lambda_{n}\right) \end{array}\right],$$

where Δ*HbO_2_*, Δ*Hb*, and Δ*oxCCO* are relative changes from baseline in oxy-hemoglobin, deoxy-hemoglobin, and the oxidation state of cytochrome c oxidase. The differential pathlength (DP) was set to 4.39 based on previous literature [32,33] and corrected for the wavelength dependency of the pathlength [34]. ΔA represents the measured change in attenuation. Relative and baseline measures of hemoglobin were combined to determine the tissue saturation as follows [27]:

(5) $$S_{t}O_{2}=\frac{\left(HbO_{2}^{b}+\Delta HbO_{2}\right)}{\left(Hb^{b}+\Delta Hb\right)+\left(HbO_{2}^{b}+\Delta HbO_{2}\right)}.$$

#### 2.4.2. DCS Analysis: Relative Measure of CBF

Acquired normalized intensity autocorrelation data were converted to electric field autocorrelation data following the Siegert relation [35,36]:

(6) $$g_{2}\left(\rho ,\tau \right)=1+\beta \frac{{\left|G_{1}\left(\rho ,\tau \right)\right|}^{2}}{{\langle I\left(\rho ,\tau \right)\rangle }^{2}}.$$

where *g*_2_(*ρ*,*τ*) is the measured normalized intensity autocorrelation, *β* is the coherence factor, *G*_1_(*ρ*,*τ*) is the electric field autocorrelation function, and <*I*(*ρ*,*τ*)> is the averaged detected intensity. The parameter ρ refers to the SDD and *τ* is the correlation time. *G*_1_ was modeled using the solution to the diffusion approximation for a semi-infinite homogenous medium and assuming the motion of light scatterers can be characterized by a pseudo-Brownian motion [35,37,38]. Fitting was conducted for ρ = 2 cm and using the dynamic *µ_a_* measurements obtained by B-NIRS. A constant value for *μ_s_*’ of 8 cm^−1^ was used in accordance with recent literature [39]. Fitting each autocorrelation curve provided an index of cerebral blood flow (CBFi).

### 2.5. Statistical Analysis

All data are presented as mean ± standard deviation (range) unless otherwise noted. Since the tapping duration varied between procedures, the perfusion, oxygen saturation, and metabolic data collected for each VT were temporally normalized to the average tap duration to obtain mean time courses. For pre- and post-VT statistics, data were limited to two minutes prior to needle insertion and following needle removal to avoid motion artifacts. To assess the overall change in CBFi, S_t_O_2_, and oxCCO due to a VT, a one-tailed paired *t*-test was used to compare pre- and post-VT measurements. Potential outliers were identified as points greater than q3 + w × (q3 − q1) or less than q1 − w × (q3 − q1) in the box plots, where w represents the whisker length and q indicates the quartile number. Statistics were repeated excluding any outliers. A matched pairs power analysis was conducted using the G*Power 3 software (v3.1.9.7, Düsseldorf, Germany) to calculate the total sample size required to achieve significance [40]. Statistical significance was based on a *p*-value < 0.05; power was set to 0.8.

## 3. Results

Optical measurements were acquired during 14 ventricular taps across four patients (one female, three males, gestational age 25.0 to 37.6 weeks, mean = 29.2 ± 5.6 weeks). Data from four events were excluded due to excessive patient motion. A further three B-NIRS datasets were excluded due to ambient light contamination. DCS data acquired during these periods were not impacted and remained in the analysis. A total of 10 ventricular taps were analyzed for CBFi, and seven for S_t_O_2_ and oxCCO. Clinical parameters are displayed in Table 1.

**Figure 3:**
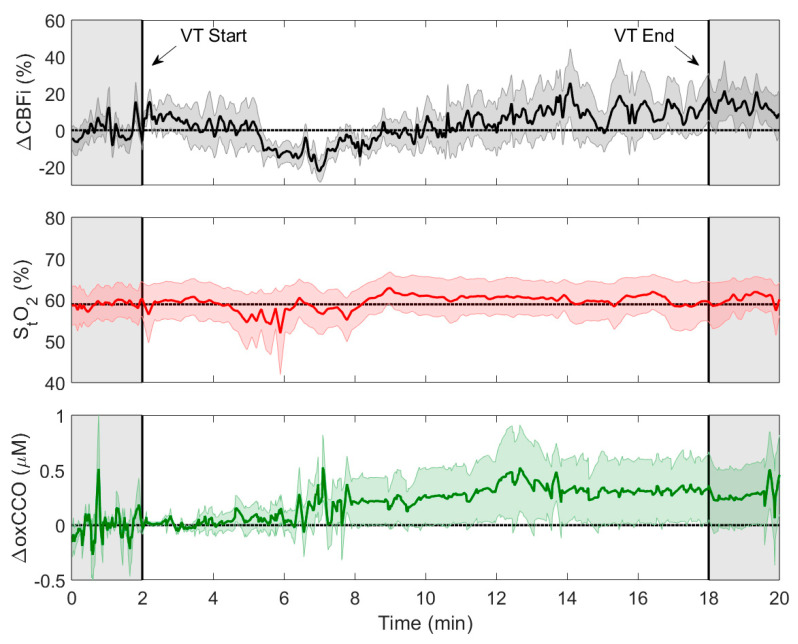
Change in cerebral blood flow index (ΔCBFi, *n* = 10), tissue saturation (S_t_O_2_, *n* = 7), and change in oxidation state of cytochrome c oxidase (ΔoxCCO, *n* = 7) averaged across multiple trials over the course of ventricular taps (VT). Data were temporally normalized to the averaged tap duration (16 min) and aligned to VT start and end points. Each time course is displayed with its standard error (shaded background).

Infants were monitored continuously throughout the ventricular tap, spanning needle insertion and CSF drainage. Ventricular tap duration varied across the interventions with an average duration of 16.1 ± 7.7 min (range: 8.75 to 29.25 min). Average time courses of CBFi, S_t_O_2_, and oxCCO during the tapping procedure are displayed in Figure 3.

**Figure 4:**
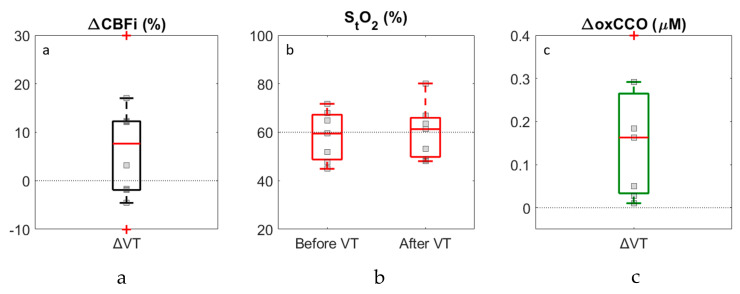
Boxplots showing changes in CBFi (**a**) and oxCCO (**c**) following ventricular tap and S_t_O_2_ (**b**) before and after the tap. Results are based on the difference between pre- and post-VT measurements that were averaged over 2-min periods. Individual data points are displayed; outliers are indicated by the crosses on the borders and represent ΔCBFi values of 122% and −25% and ΔoxCCO of 1.7 µM.

The grey shaded regions in Figure 3 surrounding the CSF drainage period represent the intervals used to calculate average pre- and post-VT values. Figure 4 presents the changes in CBFi and oxCCO relative to baseline and pre- and post-VT S_t_O_2_ values. The outliers indicated on the plot borders corresponded to ΔCBFi changes of 122.0% and −25.0%, and ΔoxCCO of 1.7 µM. Averaged values are displayed in Table 2 along with *t*-test and power analysis results. Significance was not met for any of the parameters. Power analysis indicated that sample sizes of 29 and 18 for ΔCBFi and ΔoxCCO, respectively, would be required to achieve significance. A significant increase was found for both ΔCBFi and ΔoxCCO if outliers were excluded.

## 4. Discussion

The purpose of this study was to implement the recently developed Neonatal NeuroMonitor (NNeMo) to assess cerebral hemodynamics and metabolism during ventricular taps in patients with post-hemorrhagic ventricular dilatation. Compared with commercial NIRS devices that focus on monitoring S_t_O_2_, the goal of implementing NNeMo was to assess the direct impact of a VT on CBF, as measured by DCS, and cerebral oxygen metabolism, as determined by monitoring oxCCO with B-NIRS. Both methods provide continuous recordings, which enabled CBFi and oxCCO to be monitored throughout the tapping procedure (Figure 3).

In hydrocephalus, decreases in cerebral perfusion are expected to precede changes in metabolism and clinical symptoms [41]. In agreement with this concept, reductions in CBF have been shown to impact metabolism only when CBF drops below a certain threshold [27,42]. During a VT, as the ventricle volume and ICP decrease, perfusion is expected to improve. The possibility of concurrent changes in metabolism would therefore depend on the extent to which CBF is improved by the VT. A large increase in oxCCO following a VT would suggest that the mitochondria were oxygen-limited prior to the procedure, implying that the intervention could have been performed earlier [43]. In this study, it was anticipated that a VT would improve CBF, but the magnitude of the perfusion increase would likely not be sufficient to have an effect on oxCCO. Following 10 VTs, a non-significant increase was found in ΔCBFi (14.6 ± 37.6%, *p* = 0.09) and ΔoxCCO (0.4 ± 0.6 µM, *p* = 0.09), while little change was observed in ΔS_t_O_2_ (1.9 ± 4.9%, *p* = 0.2). Power analysis indicated that achieving significance for these relatively small increases would require a sample size of 29 for ΔCBFi and 18 for ΔoxCCO. Removing the outliers in the ΔCBFi (*n* = 2) and ΔoxCCO (*n* = 1) datasets did result in significant increases in both parameters; however, the average changes remained small (6.0 ± 7.7% for ΔCBFi and 0.1 ± 0.1 µM for ΔoxCCO).

Several studies have investigated the potential impact of CSF drainage on cerebral hemodynamics in PHVD patients. An increase in CBF velocity was found by Doppler ultrasound across seven patients [44], and stable oxCCO, as measured by NIRS, in 23 infants receiving a VT or lumbar puncture (LP) [45]. In an earlier study, Casaer et al. reported that oxCCO increased in infants with grade 3 or 4 IVH who received an LP if patients had clinical signs of elevated ICP (i.e., increased head circumference, bulged fontanel, *n* = 4), but not if these symptoms were absent (*n* = 4) [46,47]. Similarly, elevated cerebral oxygenation has been reported during cerebrospinal fluid removal, which was interpreted as evidence of increased CBF [45,48,49]. Previous work by our group measured absolute CBF and the cerebral metabolic rate of oxygen (CMRO_2_) before and following a VT using dynamic contrast-enhanced NIRS [14]. A significant increase in CBF (15.6%) across 20 VTs was found with no change in CMRO_2_. In general, evidence of increased CBF following CSF removal has been consistent across studies. The lack of a metabolic response can be explained by the modest perfusion increase, indicating that the reduction in CBF prior to the VT was not sufficient to impede oxygen delivery [18,31]. The results of the current study complement these findings as the average increases in CBFi and oxCCO were small and only reached significance after removing outliers. It is important to note that the technologies used across studies to monitor cerebral perfusion and oxygen metabolism are quite different, yet the findings are similar.

Compared with McLachlan et al. [14], the use of the hybrid B-NIRS/DCS system had the advantages of not requiring an injection of an exogenous contrast agent to assess cerebral perfusion and metabolism and providing continuous monitoring of these parameters throughout the tapping procedure. The combination of B-NIRS and DCS in NNeMo was achieved using a multiplexing shutter system to prevent crosstalk between the two techniques. The intensity of the light from the DCS laser is large enough to easily saturate the B-NIRS spectrometer, which impedes its ability to detect the relatively small spectral features of oxCCO. Conversely, broadband light from the B-NIRS light source is highly incoherent, which can degrade the temporal correlation of dynamic light scattering used by DCS to measure CBFi [50]. These issues can be overcome by cycling data acquisition between the two techniques. In this study, a sample rate of 4 Hz for both systems with an overall cycling time of 7 s was considered sufficient, given the procedure was between 10 to 30 min in duration. It should be noted, however, that the multiplexing shutter system has the flexibility to achieve higher temporal resolutions if required. Both systems are capable of sampling rates up to 10 Hz, which could be used for instance to capture blood flow pulsatility [28]. The minimum cycling time would be on the order of 300 ms, accounting for shutter opening and closing times of less than 50 ms [51]. Alternately, truly simultaneous measurements could be achieved using optical filters to selectively block B-NIRS and DCS light sources from the opposing detectors. Recent works describing hybrid B-NIRS/DCS and time-resolved NIRS/DCS systems utilizing bandpass and notch filters have been proposed [50,52,53].

In this clinical application, the two major sources of signal contamination were motion artifacts and ambient light. Maintaining adequate probe contact with the scalp was challenging on infants with PHVD as the head is distended due to the enlarged ventricles. As CSF is drained during a VT, head circumference decreases, which can loosen the straps holding the probes to the head. To mitigate this issue, the probes were placed on the forehead above the frontoparietal cortex, where there is minimal change in head circumference. In addition, the elastic property of the strap used to hold the probes in place helped maintain good contact throughout the procedure. To minimize motion artifacts, an NICU nurse gently restrained the patient’s head during the VT. Other sources of motion were the repositioning of the infant in the cot for needle insertion and the application of pressure to the head after the needle was retracted (Figure 2). As a result, data analysis was limited to 2-min windows immediately prior to and following the VT. This short period prevented monitoring for possible long-term changes after the VT, despite NNeMo’s measurement stability over extended durations [27]. In three experiments, subjects were located in an environment with excessive ambient light, as reflected by greater dark count measurements acquired by B-NIRS. Since oxCCO only contributes approximately 10% of the total spectral signal [22], it was necessary to remove B-NIRS datasets in which the dark count was deemed excessive. In contrast, ambient light did not have the same impact on the DCS measurements due to the stronger intensity of the laser and therefore these datasets were included in the analysis.

Another potential source of error with optical neuromonitoring methods is the effect of scalp contamination. The influence of the scalp is considerably less in infants compared with adults, and within this study its effects were mitigated using appropriate source-detector distances for B-NIRS (SDD = 3 cm) and DCS (SDD = 2 cm) [54]. An additional source of signal contamination unique to hydrocephalus is possible light loss in the enlarged ventricle as the cortical mantle becomes increasingly thin. Our group demonstrated that this effect could distort absorption spectra as the thickness of the cortical mantle approaches 1 cm [14]. This likely did not have a significant effect on the CBFi measurements since the SDD was 2 cm. The NIRS spectra were, however, acquired with SDD = 3 cm, which would increase the chance of light loss in the ventricles. The excellent agreement in mean baseline S_t_O_2_ values from the current and previous study—58.4 ± 9.7% compared to 58.9 ± 2.7% from reference [14]—indicates that cortical thickness did not have a significant effect.

Future work will investigate a potential correlation of hemodynamic changes with subsequent markers of brain injury. Comparison to clinical outcomes has the potential to establish clinical thresholds of perfusion/metabolic change and could aid in describing VT efficacy or in gauging brain injury severity during the progression of PHVD.

## 5. Conclusions

In summary, this feasibility study presents an optical system capable of providing real-time monitoring of cerebral perfusion and oxygen metabolism in the NICU. In this application, cerebral blood flow and metabolic changes monitored throughout VTs were small and only reached significance after removing outliers. These results are in good agreement with previous studies. Considering that the hybrid optical system is portable and completely non-invasive, it is well suited for neuromonitoring in PHVD patients who may be suspected of larger cerebral hemodynamic changes based on clinical symptoms.

## Figures and Tables

**Table 1:** Clinical parameters for post-hemorrhagic ventricular dilatation (PHDV) patients.

Gestational age at birth (weeks)	29.2 ± 5.6 (25–37 4/7)
Birth weight (g)	1259.0 ± 746.7 (810–2376)
Sex (n)	F = 1, M = 3
Apgar 1 min	3.8 (1–8)
Number of VT performed (n)	7.8 (5–10)
Age at first VT (weeks)	3.8 ± 2.1 (2.3–7)
EVD (n)	2
VP shunt (n)	3
IVH Grade: Bilateral III	2
IVH Grade: III (R)/ IV (L)	1
IVH Grade: Bilateral IV	1

EVD, external ventricular drain; VP ventriculoperitoneal; IVH, intraventricular hemorrhage.

**Table 2:** Average changes following VT relative to baseline, *t*-test, and power analysis.

Statistical Parameter	ΔCBFi (%)	ΔS_t_O_2_ (%)	ΔoxCCO (µM)
Average change	14.6 ± 37.6	1.9 ± 4.9	0.4 ± 0.6
*p*-value	0.09	0.20	0.09
Required sample size for significance ^1^	29	45	18
Average change excluding outliers	6.0 ± 7.7	-	0.1 ± 0.1
*p*-value excluding outliers	0.05	-	0.02

^1^ Power analysis with G*Power 3 [40], (Power = 0.8; error probability 0.05).
